# BMSC exosome-enriched acellular fish scale scaffolds promote bone regeneration

**DOI:** 10.1186/s12951-022-01646-9

**Published:** 2022-10-12

**Authors:** Yangyufan Wang, Bin Kong, Xiang Chen, Rui Liu, Yuanjin Zhao, Zhuxiao Gu, Qing Jiang

**Affiliations:** 1 State Key Laboratory of Pharmaceutical Biotechnology, Department of Sports Medicine and Adult Reconstructive Surgery, Nanjing Drum Tower Hospital, The Affiliated Hospital of Nanjing University Medical School, 321 Zhongshan Road, 210008 Nanjing, Jiangsu PR China; 2 State Key Laboratory of Bioelectronics, School of Biological Science and Medical Engineering, Southeast University, 210096 Nanjing, China; 3 Jiangsu Key Laboratory of Molecular Medicine, Medical School, Nanjing University, 210093 Nanjing, Jiangsu PR China; 4 Branch of National Clinical Research Center for Orthopedics, Sports Medicine and Rehabilitation, Nanjing, Jiangsu China

**Keywords:** Fish scale, Exosome, Cranial defect, Osteogenesis differentiation, Tissue engineering

## Abstract

Tissue engineering scaffolds are essential for repairing bone defects. The use of biomimetic scaffolds for bone tissue engineering has been investigated for decades. To date, the trend in this area has been moved toward the construction of biomimetic acellular scaffolds with effective modification to enhance the osteogenic differentiation efficiency of bone marrow mesenchymal stem cells (BMSCs). The exosomes derived from BMSCs have been shown as a potential therapeutic tool for repairing bone defects. In this study, we demonstrated the pro-osteogenic effects of exosomes derived form osteogenic differentiated BMSCs (OBMSC) and presented a novel exosmes-functionalized decellularized fish scale (DE-FS) scaffold for promoting bone regeneration in vivo. The DE-FS scaffolds were obtained through decellularization and decalcification processes, which exhibited high biocompatibility and low immunological rejection. The intrinsic anisotropic structures of DE-FS could enhance the adhesion and proliferation ability of BMSCs in vitro. In addition, we demonstrated that the porous structure of DE-FS endowed them with the capacity to load and release exosomes to BMSCs, resulting in the enhanced osteogenic differentiation of BMSCs. Concerning these pro-osteogenic effects, it was further proved that OBMSC exosome-modified DE-FS scaffolds could effectively promote bone regeneration in the mouse calvarial defect models. In conclusion, our work provided a new insight to design exosome-riched biomimetic scaffolds for bone tissue engineering and clinical applications.

**Supplementary information:**

The online version contains supplementary material available at 10.1186/s12951-022-01646-9.

## Introduction

**Fig. 1:**
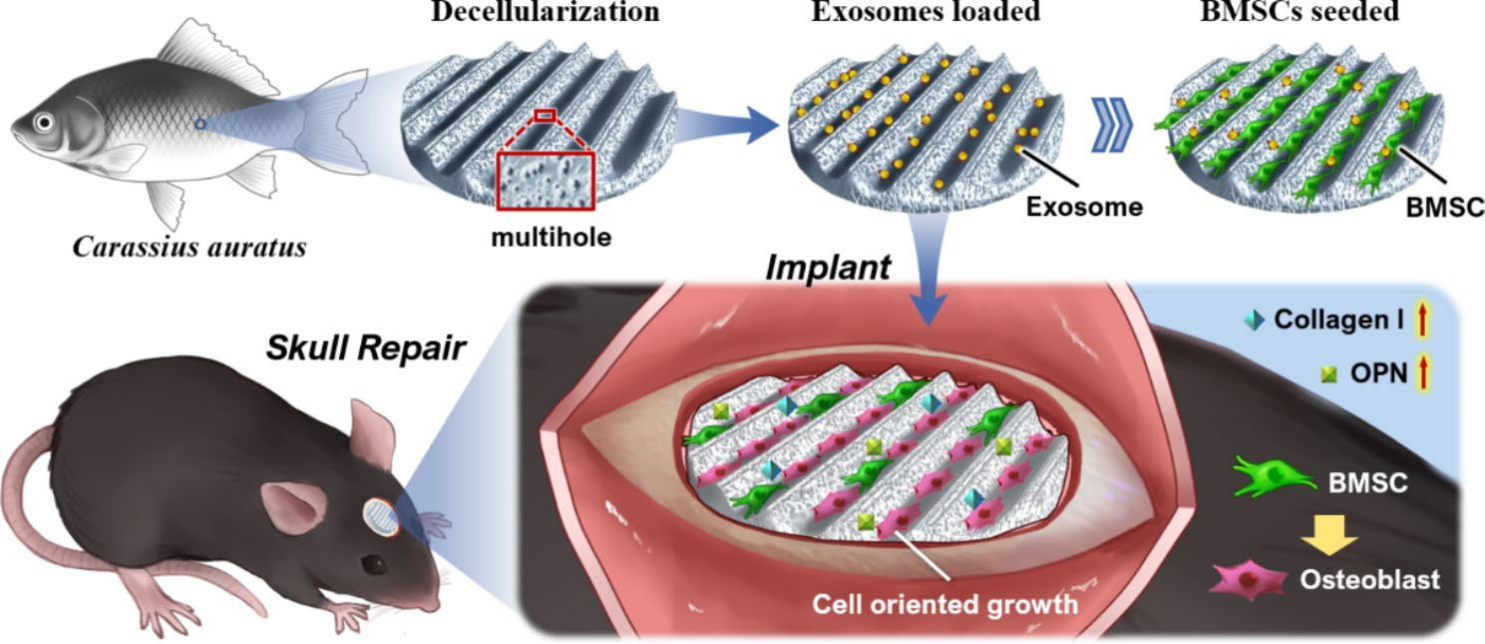
Scheme of cell-free fish scale scaffolds loaded with stem cell-derived exosomes for bone tissue regeneration

Diseases including bone infection, bone tumor-caused surgical excision, bone trauma, avascular necrosis, and osteoporosis can cause bone defects to afflict patients daily [1, 2]. Unfortunately, the limited self-healing ability of bone tissues poses a huge clinical challenge to reconstructing massive bone defects. To date, suitable materials used for bone tissue regeneration mainly include autografts, allografts, and artificial tissue engineering bone scaffolds [3–5]. Despite their extensive use in orthopedic procedures, both autografts and allografts have significant deficiencies that limit their clinical usage [6, 7]. Alternatively, artificial bone tissue engineering scaffolds can avoid the aforementioned drawbacks of using autografts and allografts, making them promising biomimetic options for patients with orthopedic disorders [8]. However, most artificial tissue engineering scaffolds are not readily accessible since multiple complex chemical synthetic steps are frequently required. In this paper, we present a novel biohybrid scaffold by integrating the advantages of decellularized fish scale (DE-FS) scaffold and osteogenic bone marrow mesenchymal stem cells (OBMSC) derived exosomes for bone regeneration (Fig. 1).

Fish scale (FS), as an efficient natural dermal armors of fish, is mainly composed of hydroxyapatite and multilayer collagen fibers to form a laminated plywood structure [9], imparting FS with excellent biocompatibility, low immunogenicity, and sufficient mechanical strength[10, 11]. In addition, its aligned fibrous structure works as functional substrates for enhanced adhesion, ordered growth, and induced differentiation of cells. In particular, the bone-repairing effects of these scaffolds could be further improved by integrating with stem cells, such as human mesenchymal stem cells (MSCs) [12]. To date, several studies have shown MSCs to be safe and efficacious in bone defect therapy via enhancing bone regeneration [13, 14]. However, cell-based therapies are impeded by several challenges in maintaining the optimal cell potency and viability during expansion, storage, and final delivery to patients [15]. In addition, implanting stem cells into such scaffolds may lead to immune rejection and undirected cell differentiation [16]. Therefore, the construction of a biomimetic acellular scaffold with effective active loading is highly anticipated.

It was reported that MSCs sectete factors involved in several cellular processes including chemotaxis, angiogenesis and osteogenesis to promote bone regeneration [17, 18]. Exosomes, the membrane-bound nanoparticles secreted by cells, can encapsulate various functional proteins, microRNAs and lipids [19, 20] and are safer than cells in terms of tumorigenicity and immunogenicity [21]. Exosomes from MSCs have been shown to facilitate bone cell proliferation and bone regeneration [16]. Moreover, these exosomes can also be added to biomaterials, enhancing their retention and stability in the injured area and thus promoting tissue repair. Therefore, integration of DE-FS with MSCs exosomes would provide a new strategy for the treatment of bone defect via enhancing bone regeneration. In our study, we isolated the exosomes from the medium of osteogenic differentiated BMSCs that termed as OBMSC-Exos, which could induce the osteogenic differentiation of BMSCs in vitro. Concerning the pro-osteogenic effects of OBMSC-Exos, we further established a desired scaffold via encapsulating OBMSC-Exos in DE-FS, which could sustainably release exosomes and promote the osteogenic differentiation of BMSCs. The effects of OBMSC-Exos-loaded DE-FS scaffolds on osteogenesis and efficiency in forming large-scaled bone tissue were further investigated in vivo. With the characterization of such osteoinductivity, the exosome-modified DE-FS scaffold would provide a new avenues in bone defects regeneration.

## Materials and methods

### Decellularization of fish scales

The FS isolated from *Carassius auratus* was washed in water five times and then decellularized according to the method described previously [22]. Briefly, the FS was first treated with 0.1% EDTA (Sigma) and 10 mM Tris-HCl buffer (THB, Sigma). Next, the cellular component in the FS was removed using THB containing 0.1% SDS (Sigma) at 4℃ for 3 days. Lastly, the FS was sterilized with 75% ethanol and stored in 50 ml sterilized phosphate buffer saline (PBS, pH 7.4) at 4℃ for further application.

### Scanning electron microscopy (SEM)

To identify the microscopic structure, the DE-FS was fixed on a carbon-coated copper grid and dried by a mercury lamp for 5 min. Next, the surface morphology of DE-FS was imaged with SEM (Hitachi, TM-1000, Japan). The scaffold was fixed in paraformaldehyde and sequentially dehydrated with different concentrations of ethanol for 30 min for each concentration to observe the morphology of BMSCs seeded on the DE-FS surface. Finally, after freeze-drying, the samples were observed with SEM.

### Cytotoxicity and proliferation

For the cellular cytotoxicity assay, BMSCs were suspended in DMEM (WISENT, Nanjing, China) and seeded on the surface of DE-FS (n = 6) for 24 h, followed by treatment with 5 mg/ml propidium iodide (PI) and 4 mg/ml Calcein-AM in the dark and examined under a fluorescence microscope (Nikon, Tokyo, Japan). For cell viability and cell growth assay, BMSCs were cultured on the surface of DE-FS or cell culture plate (n = 6) for 1, 2, 3 and 5 days, followed with the incubation with Cell counting kit 8 (CCK-8) solution for 2 h at 37 °C until the color turns orange. Then, the results were recorded by measuring absorbance at 450 nm. In addition, cell proliferation ability was further determined by EdU staining (Invitrogen). Briefly, BMSCs were cultured on the surface of DE-FS or cell culture plate (n = 6) for 24 h, and further incubated with 10 μm EdU solution for 2 h to incorporate Alexa Flour 488-labeled EdU into newly synthesized DNA. The results were imaged by confocal fluorescence microscope (Olympus).

### Osteogenic differentiation assay

To investigate osteogenic differentiation, BMSCs were cultured on DE-FS (n = 6 each group) loaded with or without exosomes supplemented with osteogenic media for 2 weeks. Then, the cells underwent 2 times of PBS washing, followed by 4% paraformaldehyde fixation for 5 min, and incubation with 2% Alizarin Red S (ARS) solution (Sigma) for 30 min. Finally imaged using a microscope (Nikon, Tokyo, Japan). To quantify the calcium amount on the DE-FS, the red matrix precipitate was solubilized in 10% cetylpyridinium chloride (Sigma). The eluted stain was read at 562 nm. In addition, alkaline phosphatase (ALP) activity of BMSCs that cultured on DE-FS (n = 6) loaded with or without exosomes undergo differentiation by incubation in osteogenic medium for 7 days was detected using an ALP Activity Assay Kit (Elabscience, Wuhan, China). The wavelength of 520 nm was set for detection in microplate reader. The data was normalized to the total protein contents determined by a BCA Protein Colorimetric Assay Kit (Elabscience).

### Western blot

BMSCs or exosomes were lysed by RIPA buffer (Beyotime, Shanghai, China). The isolated proteins were then further transferred to a PVDF membrane (Millipore), which was further blocked by non-fat milk before incubation with primary antibodies, including anti-CD63 (Abcam, ab193349, 1:1000 dilution), anti-TSG101 (Abcam, ab125011, 1:1000 dilution), anti-RUNX2 (Abcam, ab236629, 1:1000 dilution), anti-Collagen I (Abcam, ab260043, 1:1000 dilution), anti-OPN (Abcam, ab283656, 1:1000 dilution) and anti-GAPDH (ABclonal, AC002, 1:1000 dilution) overnight, and corresponding secondary antibodies for 1 h. Finally, the membrane was washed with PBST and reacted with a chemiluminescent substrate (Thermo Scientific, Waltham, MA, USA). The band was imaged by a Tanon 5200 Multi Scanning System. This experiment was performed in triplicate.

### Quantitative PCR (qPCR)

Total RNA was extracted from cultured cells using Trizol reagent (Invitrogen). Then, the cDNA was synthesized using a HiScript 1st Strand cDNA Synthesis Kit (R111-01, Vazyme, Nanjing, China) according to the manufacturer’s instruction. Finally, qPCR was performed using the ChamQ Universal SYBR qPCR Master Mix (Q711-02, Vazyme). The primers were listed in the supplementary Table 1. The levels of genes of interest were normalized to GAPDH using the 2^−ΔΔCt^ method.

### Immunofluorescence staining

BMSCs cultured on the surface of DE-FS were washed with PBS twice. Then, cells were immersed in 0.5% Triton-X 100 (Solarbio, Beijing, China) and stained with FITC-labeled Phalloidin (Beyotime). The nuclei were stained with DAPI (Beyotime). The images was observed under a fluorescence microscope (Olympus).

### Subcutaneous implantation

The DE-FS was implanted into a subcutaneous space (1 cm-long incision in the right, high dorsal flank) in the C57BL/6 mouse (n = 6 each group). In Week 3 after implantation, the mice were sacrificed with their whole blood, subcutaneous tissues, hearts, livers, spleens, lungs and kidneys obtained. Whole blood was for biochemical analysis. Tissues fixed with formalin were sliced and stained with hematoxylin & eosin (H&E).

### Preparation of osteogenic BMSC-derived exosomes

The osteogenic BMSC-derived exosomes were collected using the conventional density gradient ultracentrifugation method. In brief, BMSCs were cultured under osteogenic differentiating media with 10% exosome-free FBS (Gibco). After 7 days, the supernatant of the medium was centrifuged at 3000 g for 10 min to remove cells and debris, followed with 10,000 g centrifugation for 30 min and 100,000 g for 70 min to separate exosomes. All procedures were performed at 4 °C. The pellet was finally suspended in PBS. The size distribution of exosomes was measured by Nanoparticle tracking analysis. A particle size distribution was created with estimated particle diameter on x-axis and concentration on the y-axis. Western blot assay was further performed to verify the presence of exosomes.

### Transmission electron microscopy (TEM)

Freshly isolated exosomes were fixed in glutaraldehyde overnight. Then, the suspension of exosomes was loaded onto copper mesh Formvar-coated grids. For negative staining of exosomes, the grids were applied with aqueous phosphotungstic acid for 60 s. The grids were then allowed to dry and imaged with a TEM (H7500) operated at 80 kV.

### Exosome uptake assay

For uptake studies, the isolated exosomes were stained with a red fluorescent dye (PKH26, Sigma-Aldrich, USA) according to the manufacturer’s protocol. Briefly, the exosome pellet was resuspended in 1 mL Diluent C, adding with 4 µl PKH26 dye. After incubation for 4 min at room temperature, 2 mL FBS were added to bind the excess dye. The labeled exosomes were collected by centrifuging at 100,000 g for 1 h, which were further loaded on the DE-FS. Then, BMSCs were cultured on the surface of DE-FS. The uptake of exosomes were visualized with a laser scanning confocal microscope (Olympus).

### Loading and releasing exosomes on the DE-FS

Exosomes with a density of 5 × 10^9^ per mL were incubated with DE-FS (n = 6) at 4 °C for 12, 24, and 48 h. The loading efficiency was calculated by dividing the number of initial exosomes by the exosomes loaded on the DE-FS. The exosome release from DE-FS was performed in a basal medium at 12, 24, and 48 h. The release efficiency was calculated by dividing the number of exosomes loaded on DE-FS by that in the supernatant.

### Calvarial defect model

The animal experiment approval was granted by the Animal Research Committee of Nanjing Drum Tower Hospital. Eight-week-old C57BL/6 mice (n = 6 each group) bought from Ziyuan Biotechnology (Hangzhou, China) were kept according to the Guidelines. After isoflurane anesthetization, a calvarial defect with a thickness of 3 mm was made at the parietal bone. The dura mater was preserved from injury in the procedure. A 3-mm scale exosome-laden DE-FS was implanted in each defect region. DE-FS without OBMSC-derived exosomes was used as the control. After full recovery from anesthesia, the mice were sent to the vivarium for the subsequent procedure.

### Microcomputerized tomography (µCT) scanning

At week 8 after the surgery, the mouse calvarial bone was collected and fixed in formaldehyde for 1 day. The sample was stored at 75% ethanol and later scanned by a high-resolution MicroCT scanner (SkyScan, Kontich, Belgium) at 57 kVp, 184 µA, 0.5 mm filtration, and 10 μm resolution. Reconstruction of 3D images was acquired with Dolphin 3D software e (Dolphin Imaging & Management Solutions, CA, USA). The bone volume/tissue volume (BV/TV%) and trabecular number (Tb.N.) were calculated using the CTan software. The growth area of relative bone (bone growth area %) was obtained using ImageJ software.

### Histological analysis

After µCT scanning, the fixed calvarial bone tissue was decalcified with EDTA solution, gently shaken for up to 2 weeks, then dehydrated, transparentized, and sliced. H&E, and Masson were carried out and imaged by an Olympus IX71 microscope. Immunohistochemical staining for F4/80 was performed as follows. The sections of mouse skin were deparaffinized in xylene, and rehydrated in alcohols. Then, the samples were treated with citrate buffer for 20 min at 100 °C for epitope retrieval. The endogenous peroxidase was further quenched with 3% H_2_O_2_. The skin sections were blocked with goat serum and incubated with F4/80 antibody (SAF002, AiFang biological, Changsha, China) overnight at 4 °C. The following day, slides were incubated with secondary antibody and detected by using a DAB peroxidase substrated kit (Beyotime, Shanghai, China). Sections were imaged and photographed using an Olympus IX71 microscope.

### Statistical analysis

Quantitative data were presented as the mean ± standard error, with *p < 0.05 and **p < 0.01 indicating statistical significance. One-way analysis of variance (ANOVA) with Tukey’s post-hoc test was adopted to compare more than two groups and the two-tailed Student’s *t*-test for between-group comparison.

## Results and discussion

### Characterization of DE-FS

**Fig. 2:**
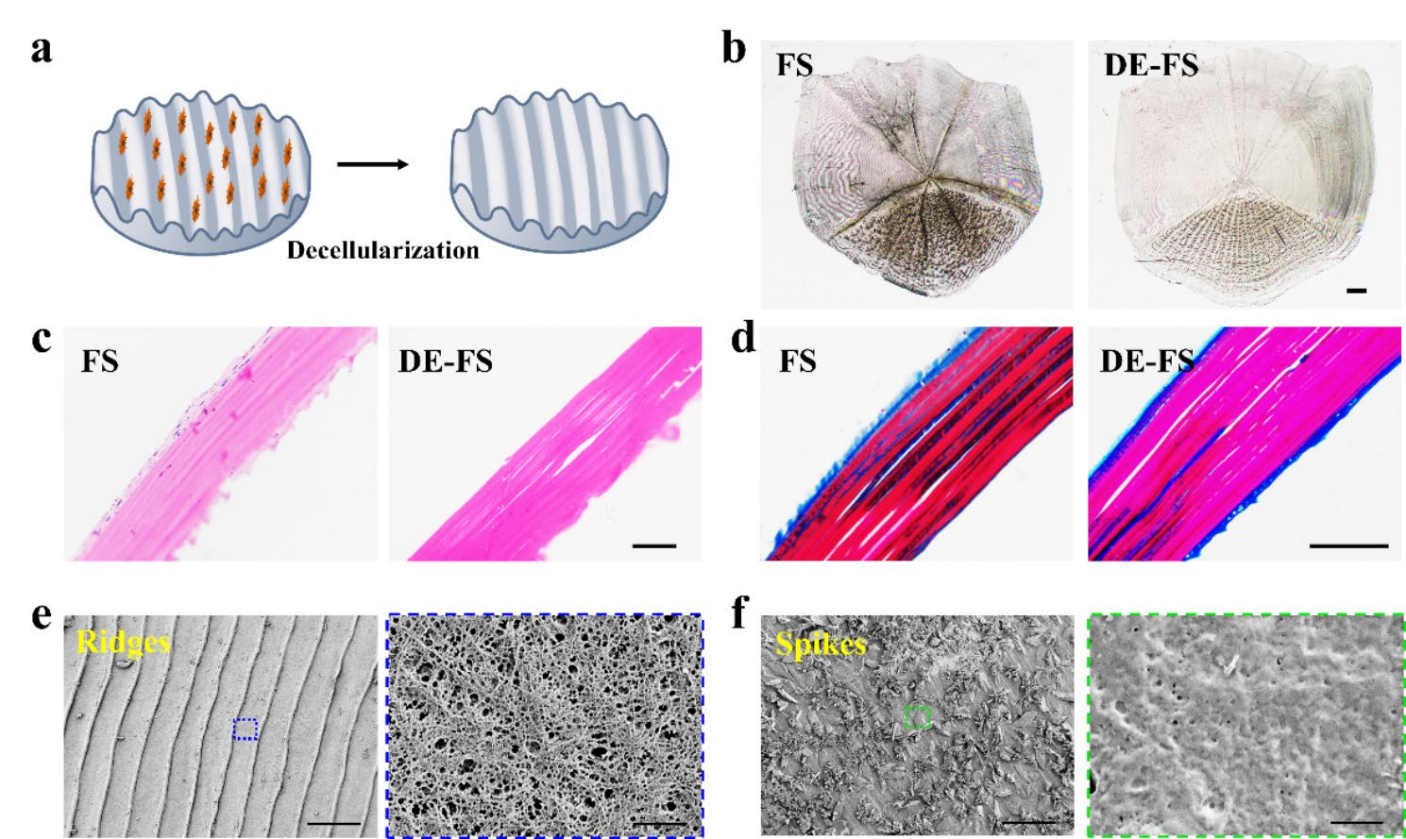
Characterization of decellularized fish scale. (a) The schematic diagram of FS before and after decellularization. (b-d) The morphology and histological staining of the FS before and after decellularization. Scale bar was 1 mm (b) and 100 μm (c, d). (e, f) SEM images of the ridges and spikes region of FS after decellularization. Scale bar was 100 μm (left panel) and 1 μm (right panel)

Massive bone defect reconstruction is challenging for clinicians. Tissue engineering, which mainly consists of biomaterial scaffolds, cells and growth factors, has been widely investigated to induce injured tissue regeneration, including defective bones [23, 24]. In our work, the DE-FS was adopted as the scaffold for the regeneration of bone tissues. FS was decellularized((Fig. 2a, b), H&E and Masson staining(Fig. 2c, d) were conducted to confirm decellularization degree. As shown in Fig. 2c and d, cellular components of the FS were successfully removed, and Masson staining demonstrated that the surface of DE-FS was coated with collagen fibers without structural changes. The presence of collagen within the implanted materials has been reported beneficial to cell adhesion, growth and differentiation of BMSC to osteoblast, thereby promoting bone defect repair [25]. In addition, SEM showed that the ridge surface of DE-FS was highly organized (approximately 55 μm of each interval) with a porous uniform microstructure (Fig. 2e). The average pore size was approximately 150 nm, similar to the diameters of exosomes (Fig. 2e). Highly porous scaffolds for the loading of exosomes have been demonstrated to exert therapeutic effects constantly and prevent the premature clearance of exosomes [26, 27]. Thus, the natural porous microstructure of DE-FS may provide an ideal platform for the encapsulation and delivery of exosomes, which would serve as a potential bone regeneration scaffold.

### Biocompatibility evaluation of DE-FS

**Fig. 3:**
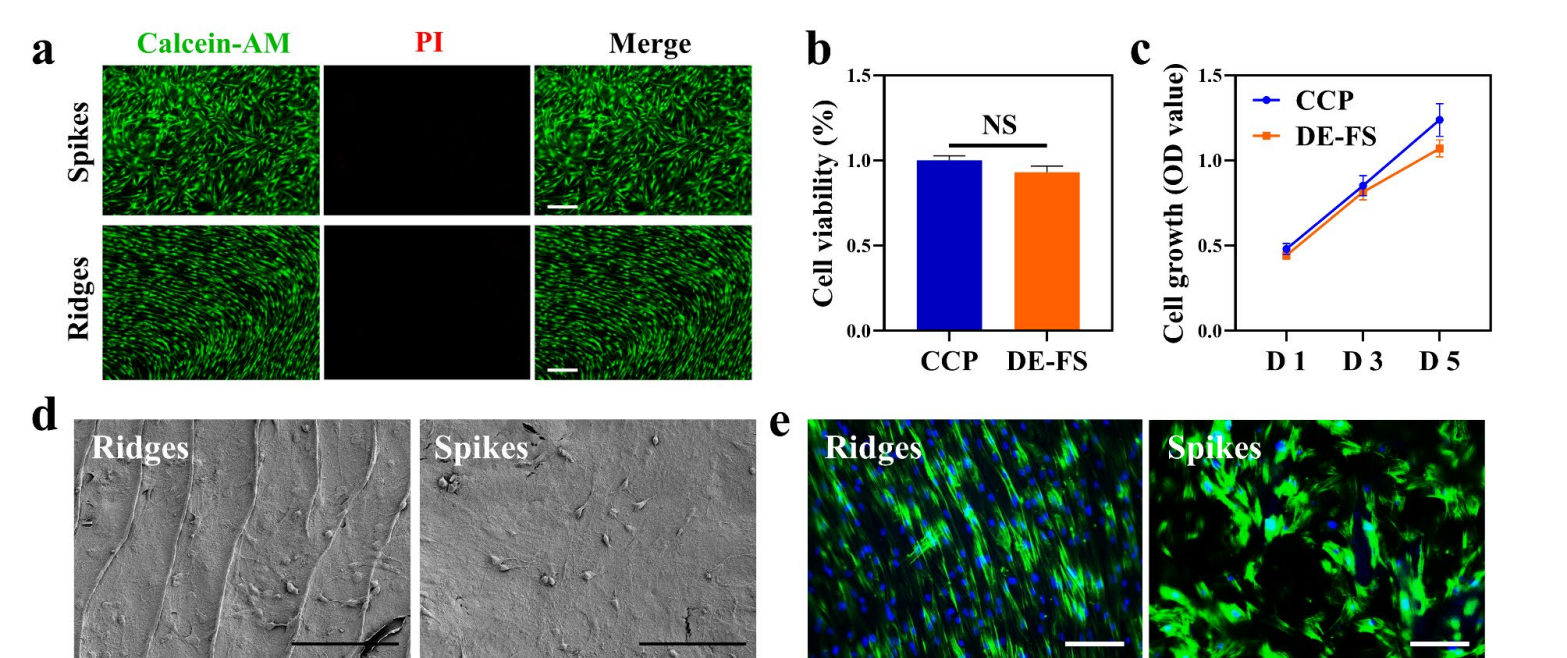
Evaluation of the biocompatibility of the decellularized FS. (a) FDA/PI staining of BMSCs cultured on the ridge and spike regions of decellularized FS. Scale bar was 500 μm (b) The cell viability of BMSCs cultured on a cell culture plate (CCP) or decellularized FS for 48 h. (c) The cell growth of BMSCs cultured on CCP or FS. (d, e) SEM images and phalloidin staining of BMSCs cultured on the ridge and spike regions of decellularized FS. Scale bar was 100 μm

**Fig. 4:**
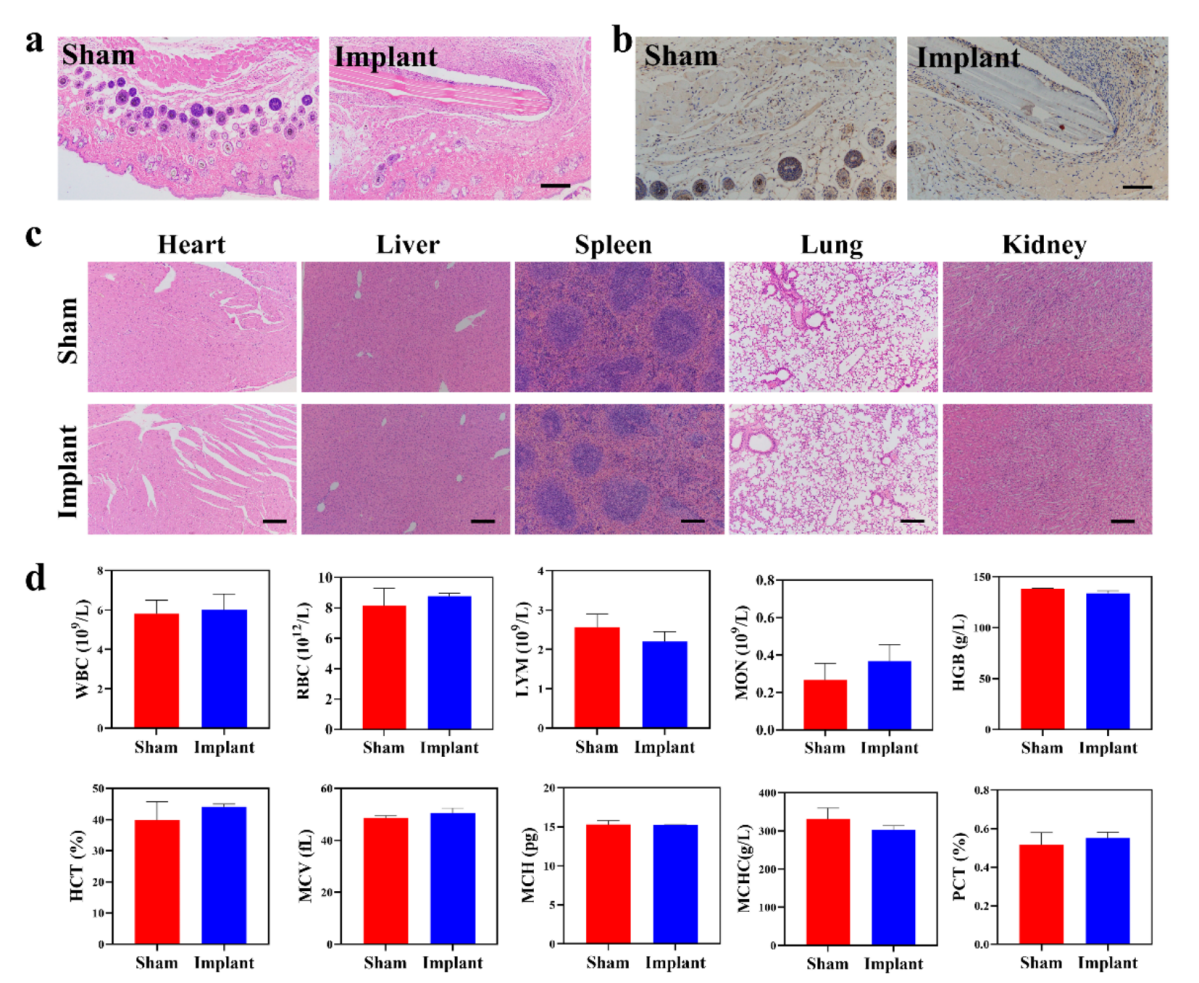
In vivo biosafety evaluation. (a, b) H&E and immunohistochemical of F4/80 staining of the skin from mice implanted with or without DE-FS for 2 weeks. Scale bar was 400 μm. (c) H&E-stained samples obtained from the heart, liver, spleen, lung, and kidney of mice implanted with or without DE-FS for 2 weeks. Scale bar was 400 μm. (d) Blood biochemistry analysis of mice after implantation with or without DE-FS for 2 weeks with examined parameters including white blood cell (WBC) count, red blood cell (RBC) count, lymphocyte (LYM), monocyte (MON), hemoglobin (HGB), hematocrit (HCT), mean corpuscular volume (MCV), mean corpuscular hemoglobin (MCH), mean corpuscular hemoglobin concentration (MCHC), and platelets (PLT).

Generally, scaffolds for tissue engineering should be biocompatible without high cytotoxicity and strong inflammatory response after implantation into the host. To verify the biocompatibility of the DE-FS in vitro, human BMSCs were seeded on the surface of the DE-FS. FDA/PI staining of BMSCs after the 3-day incubation indicated good adhesion of BMSCs to the DE-FS surface, with few apoptotic cells (Fig. 3a). The CCK-8 and EdU assay also demonstrated normal growth and proliferation of BMSCs on the DE-FS surface compared with those cultured on cell culture plates (Fig. 3b, c, S1). SEM images showed that human BMSCs of spindle shapes spread on the ridges and spikes of DE-FS (Fig. 3d). Interestingly, we found that BMSCs grew orderly along the ridges in the ridge area, while disorderly growth was observed in the spike area (Fig. 3e). The immunogenicity of DE-FS was detected by a classical challenge test on mice. Subcutaneous DE-FS implantation in the dorsal flank of mice (n = 6) was conducted to evaluate the tissue response by comparison with the sham group. Then, histological analysis and a complete blood panel were performed to evaluate biosafety in vivo. H&E and immunohistochemical staining of the DE-FS surrounding the subcutaneous tissues at Week 3 after implantation showed no pathological change or enhanced macrophage infiltration compared with the sham group (Fig. 4a, b). The H&E staining of other tissues also had no pathological changes compared to the sham group (Fig. 4c). Besides, complete blood panel demonstrated no significant difference in immune response at Week 3 after DE-FS implantation compared with the sham group (Fig. 4d). Together, our results indicated that DE-FS had high biocompatibility and thus could potentially be used as a biomimetic scaffold.

### Characterization of OBMSC-derived exosomes

**Fig. 5:**
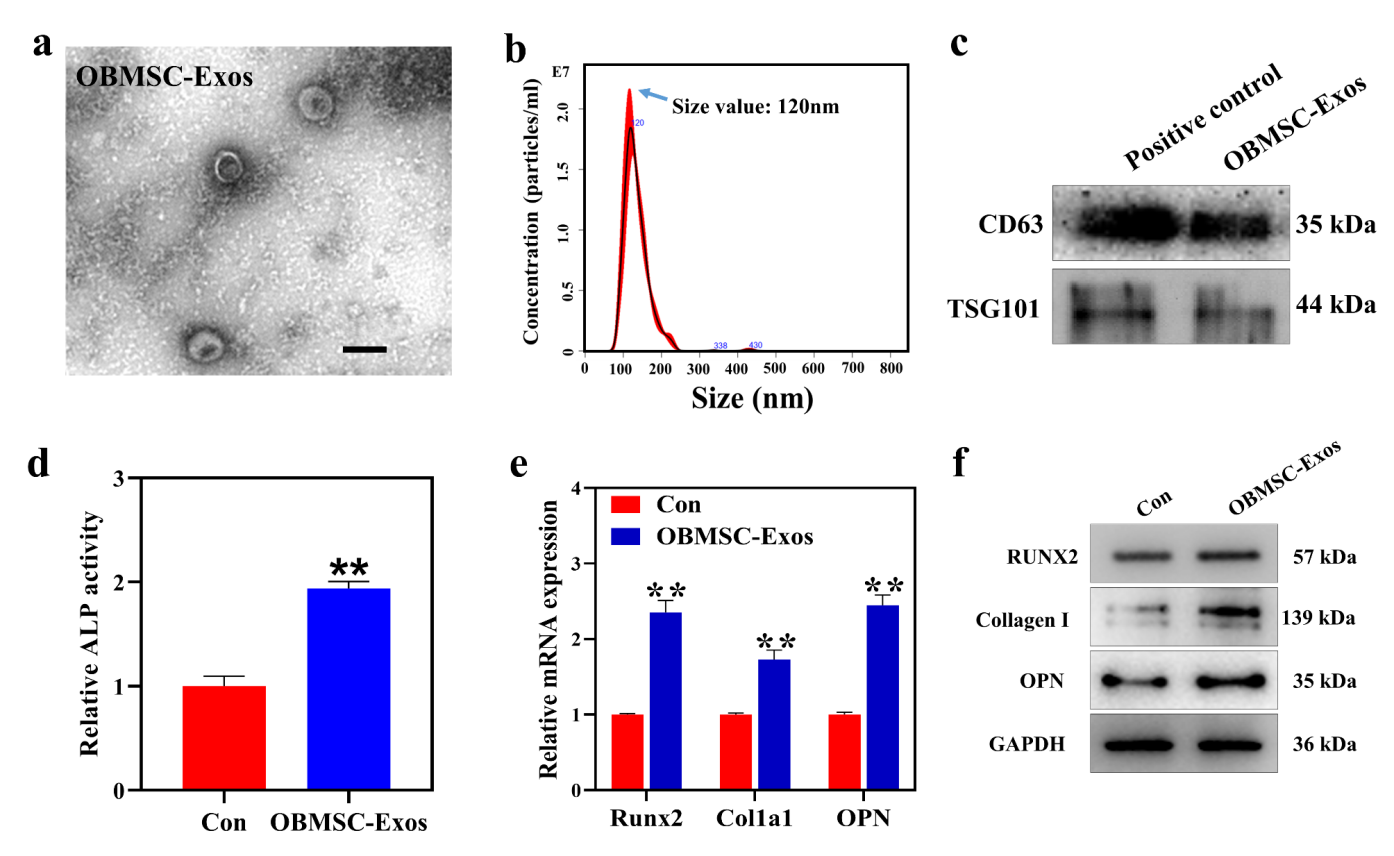
Enhanced osteogenic induction by OBMSC-Exos in vitro. (a, b) The size distribution of the microvesicle fraction isolated from osteogenic OBMSCs. Scale bar was 100 nm. (c) Western blot results of the expression of specific markers of exosomes (CD63 and TSG101). (d) Relative ALP activity of BMSCs treated with or without OBMSC-Exos for 7 days. (e) Q-PCR analysis of Runx2, Col1a1 and OPN expression in BMSCs treated with or without OBMSC-Exos for 7 days. (f) Western blot analysis of RUNX2, Collagen I and OPN expression in BMSCs treated with or without OBMSC-Exos for 7 days

Recently, majority of researchers have devoted to application of MSCs in bone regeneration. It was widely accepted that the MSCs promoted the regeneration and repair through releasing growth factors, cytokines and other extracellular molecules to modulate endogenous cell migration and differentiation [28]. It was reported that the conditioned medium of MSCs exerts a beneficial role in bone regeneration, as the secretome participates in multiple cellular functions [29]. Among the numerous trophic factors secreted by MSCs, MSCs exosomes were reported to be efficacious in enhancing bone regeneration [30]. Increasingly, MSCs exosomes have replicated the wide-ranging therapeutic efficacies of MSCs in tissue injury [31]. Hence, cell-free tissue engineering is being vastly investigated as a safer and more effective strategy than the traditional cell-based therapy due to its acellular nature. In this work, we isolated exosomes from the supernatant of BMSCs pretreated with osteogenic differentiation medium via density gradient ultracentrifugation. TEM revealed that the osteogenic BMSC-derived exosomes (OBMSC-Exos) were dense with a solid shape, a typical double-layered membrane structure, and an average diameter of approximately 110 nm (Fig. 5a). Nanoparticle tracking analysis (NTA) also indicated that the purified exosomes were indeed nearly spherical nanoparticles with diameters of approximately 135.8 ± 3.8 nm, which was in line with our TEM findings (Fig. 5b). Moreover, in the detection of exosome marker proteins (CD63, TSG101) by using Western blot analysis, we found that exosomes isolated from osteogenic BMSCs supernatant expressed CD63 and TSG101 as compared to the positive control (Fig. 5c), which confirmed the identity of exosomes. To further assess the influence of exosomes on the osteogenic differentiation of BMSCs in vitro, BMSCs were treated with or without OBMSC-Exos for 7 days. OBMSC-Exos markedly enhanced the ALP activity (Fig. 5d) and elevated the levels of RUNX2, Collagen I and OPN in BMSCs, as compared with the control group (Fig. 5e, f), suggesting that OBMSC-Exos could enhance the osteogenic differentiation of BMSCs.

**Encapsulation of OBMSC-Exos on DE-FS*in vitro***.

**Fig. 6:**
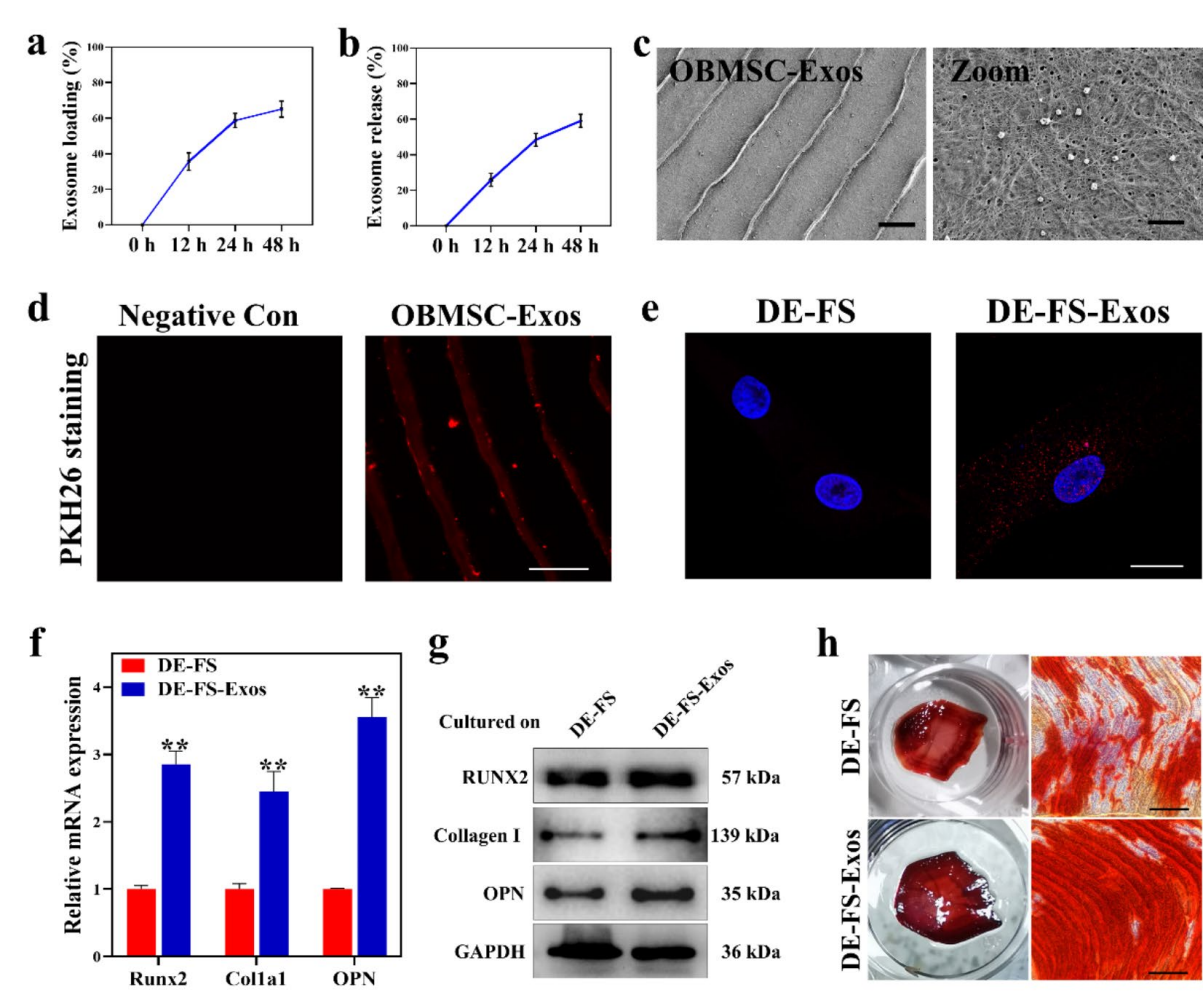
Enhanced osteogenesis of DE-FS with OBMSC-Exos encapsulation in vitro. (a) The exosome loading efficiency of DE-FS as determined by detecting the concentration of exosomes in the medium. (b) Exosomes released from DE-FS loaded with exosomes were determined by detecting the concentration of exosomes in the basal medium. (c) SEM images of DE-FS cultured with OBMSC-Exos for 24 h. Scale bar was 20 μm (left panel) and 2 μm (right panel). (d) Confocal images of DE-FS cultured with PKH26-stained OBMSC-Exos. Scale bar was 20 μm. (e) Confocal images of BMSCs cocultured with DE-FS treated as in Fig. 5D. (f) Q-PCR analysis of Runx2, Col1a1 and OPN expression in BMSCs cultured on the surface of DE-FS with OBMSC-Exos encapsulation. (g) Western blot analysis of RUNX2, Collagen I and OPN expression in BMSCs cultured on the surface of DE-FS with OBMSC-Exos encapsulation. (h) Alizarin red staining of BMSCs cultured on the surface of DE-FS with or without OBMSC-Exos encapsulation. Scale bar was 200 μm

It was known that scaffold plays a vital role in bone defect regeneration by supplying a supporting environment for cells and serving as growth factor carriers with the ability of controllable release to induce bone tissue growth during repair[32–34]. Considering the biocompatibility of DE-FS and the osteogenetic effects of OBMSC-Exos, we investigated the potential of OBMSC-Exos-loaded DE-FS for the osteogenetic differentiation of BMSCs in vitro. First, we measured the loading and release efficiency of OBMSC-Exos on the DE-FS scaffolds. After the 24-hour incubation, approximately 60% of exosomes were loaded on the DE-FS, and the loading efficiency decreased in the following 24 h (Fig. 6a).The exosome was persistently released from the scaffold after loading, with the release efficiency reaching 50% after 24 h (Fig. 6b). To confirm whether the OBMSC-Exos could be loaded on the surface of DE-FS, the OBMSC-Exos were inoculated on the DE-FS overnight, and the SEM images showed that OBMSC-Exos could attach to the DE-FS surface (Fig. 6c). We also employed PHK26, a dye that could bind to the membrane of exosomes for further validation. After the 24-hour incubation with DE-FS, we found numerous red fluorescence puncta on the DE-FS surface, indicating the successful loading of OBMSC-Exos (Fig. 6d). In addition, fluorescence staining demonstrated that the exosomes loaded on DE-FS were released and then taken up by BMSCs cultured on the surface of DE-FS (Fig. 6e). Altogether, these results suggested the successful loading of OBMSC-Exos on DE-FS and their continuous release into the surroundings. Furthermore, BMSCs cultured on DE-FS surface loaded with OBMSC-Exos showed higher expressions of Collagen I, RUNX2, and OPN (Fig. 6f, g), elevated ALP activity (Fig. S2) and increased deposition of calcium than those cultured on DE-FS without loading (Fig. 6 h, S3), indicating that the encapsulation of OBMSC-Exos on DE-FS could enhance the osteogenesis of BMSCs, which might be used as a therapy of bone defects.

**DE-FS loaded with OBMSC-Exos for calvarial bone healing*in vivo***.

**Fig. 7:**
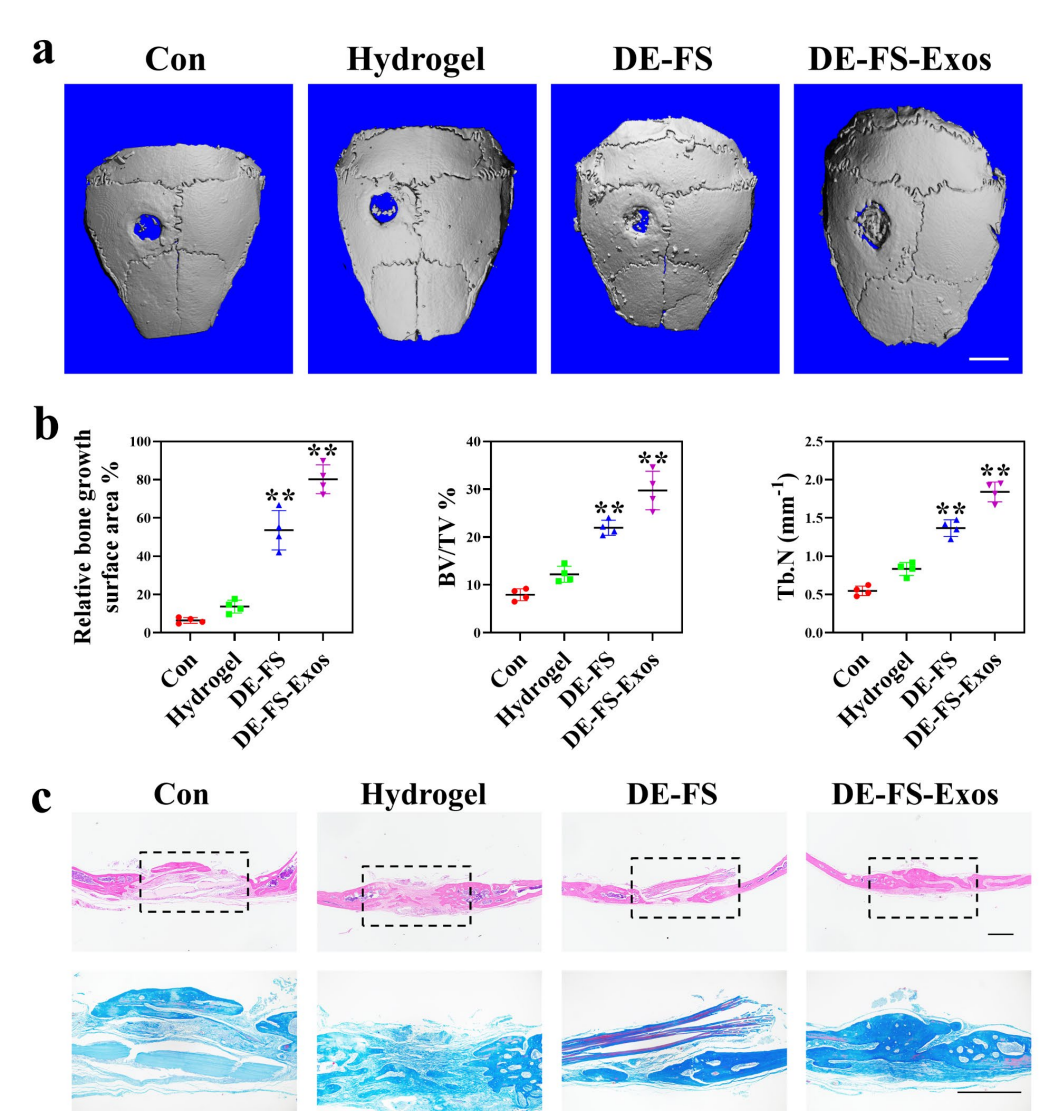
DE-FS loaded with OBMSC-Exos for calvarial bone healing in vivo. At Week 12 after the operation, the implants collected from mice underwent microCT scanning, together with quantitative and histological analyses. (a) Representative 3D reconstruction microCT images of calvarial bone defects in mice at Week 12 after the implantation of scaffolds. Scale bar was 2 mm (b) Quantitative analysis of new bone area, bone volume/tissue volume (BV/TV) and trabecular number (Tb.N). (c) H&E and Masson staining of calvarial bone defects from mice at Week 12 after the implantation of scaffolds. Scale bar was 500 μm

According to the in vitro results, we further investigated the efficacy of OBMSC-Exo-loaded DE-FS in bone repair in vivo. The reconstructed 3D micro-CT images of mice with calvarial defects implanted with OBMSC-Exos-loaded DE-FS or DE-FS for 12 weeks are shown in Fig. 7a. Micro-CT analysis demonstrated that the implantation of OBMSC-Exos-loaded DE-FS induced more marked bone healing than the exosome-free DE-FS or the control. The OBMSC-Exos-loaded DE-FS showed almost full bone healing, whereas the others only showed partial bone healing. Quantitative analysis of the micro-CT scan exhibited about 80% bone healing in OBMSC-Exos-loaded DE-FS, while only 6% in the control and 53% in exosome-free DE-FS (Fig. 7b). Apart from bone volume, tone microarchitecture is also pivotal to the mechanical strength of trabecular bone [35]. Therefore, structural indexes like trabecular number or thickness are commonly employed to evaluate the quality of regenerated bone. In this study, a significantly increased trabecular number was found in OBMSC-Exo-loaded DE-FS (Fig. 7b). In addition, we further confirmed this finding via histological examination. H&E and Masson staining results indicated that defects treated with OBMSC-Exos-loaded DE-FS presented large amounts of new bone regeneration in comparison with the control, suggesting that DE-FS loaded with OBMSC-Exos could effectively promote the remodeling of the bone structure after injury (Fig. 7c). These in vivo results showed that OBMSC-Exos-loaded DE-FS could trigger substantial bone healing in a non-healing model of mouse calvarial defects. Compared to the DE-FS scaffolds without exosome loading, the OBMSC-Exos-loaded DE-FS scaffold displayed augmented bone regeneration. The effectiveness in inducing bone repair in the early restoration stage and the molecular mechanism underlying osteogenesis of this system need in-depth investigation in the future.

## Conclusion

In the study, we proposed a novel approach based on exosome-modified DE-FS to promote the regeneration of bone, expand our understanding of the potential of natural biological materials in bone regeneration. Here, we demonstrated the high biocompatibility of DE-FS both in vitro and in vivo, which enabled osteogenic differentiation of BMSCs by uploading exosomes isolated from the supernatant of BMSCs pretreated with osteogenic differentiation medium. As a promising scaffold material, DE-FS loaded with exosomes brings inspirations and possibilities for bone repair.

## Electronic supplementary material

Below is the link to the electronic supplementary material.

Supplementary Material 1

## Data Availability

The data that support the findings of this study are available from the corresponding author upon reasonable request.

## Declarations

### Authors’ information

wangyangyufan@163.com, kongbin13@163.com, chenxiang910110@163.com, liur16_thu@foxmail.com, gzx@seu.edu.cn, yjzhao@seu.edu.cn, qingj@nju.edu.cn.
