# Supplementary material for: BMSC exosome-enriched acellular fish scale scaffolds promote bone regeneration
Source: J Nanobiotechnology. 2022 Oct 12;20:444. doi: 10.1186/s12951-022-01646-9 (PMC9555002; doi:10.1186/s12951-022-01646-9)
Supplement: Supplementary file 1 — Supplementary Material 1 [file 12951_2022_1646_MOESM1_ESM.docx]

**Supplementary materials**

**Table 1. RT-PCR primers and products.**

| Genes | Forward primer | Reverse primer |
| --- | --- | --- |
| Runx2 | AGAGTCAGATTACAGATCCCAGG | TGGCTCTTCTTACTGAGAGAGG |
| Col1a1 | TGGCTCTTCTTACTGAGAGAGG | CCACGTCTCACCATTGGGG |
| OPN | ATGGCCCAAAGGCTTACAGG | CCACAAGAATCATCCAGGTGC |
| GAPDH | CCACAAGAATCATCCAGGTGC | CCACAAGAATCATCCAGGTGC |


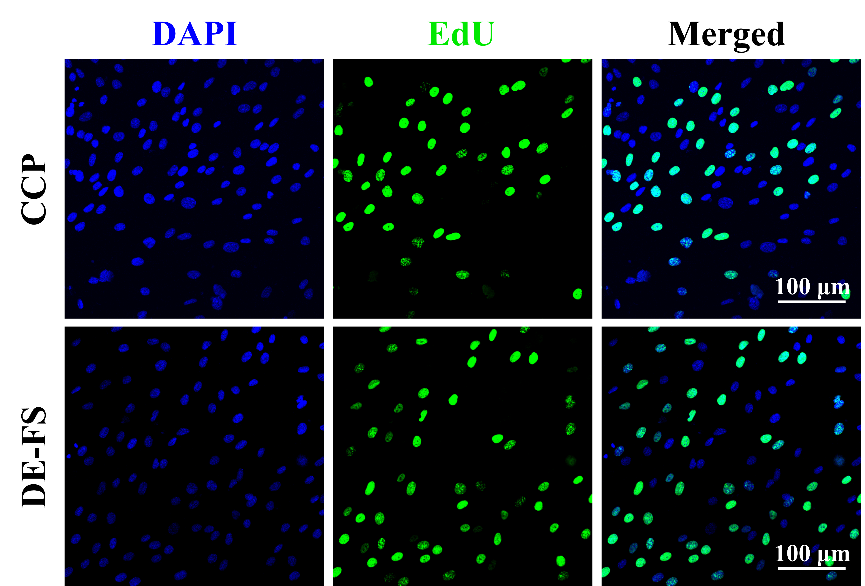


**Figure S1.** The proliferation ability of BMSCs cultured on cell culture plate or DE-FS.

**Figure S2.** The ALP activity of BMSCs cultured on the surface of DE-FS with or without OBMSC-Exos encapsulation.

**Figure S3.** Quantification of Alizarin red staining of BMSCs cultured on the surface of DE-FS with or without OBMSC-Exos encapsulation.
